# Overexpression of p-Akt, p-mTOR and p-eIF4E proteins associates with metastasis and unfavorable prognosis in non-small cell lung cancer

**DOI:** 10.1371/journal.pone.0227768

**Published:** 2020-02-05

**Authors:** Junmi Lu, Hongjing Zang, Hongmei Zheng, Yuting Zhan, Yang Yang, Yuting Zhang, Sile Liu, Juan Feng, Qiuyuan Wen, Mengping Long, Songqing Fan

**Affiliations:** Department of Pathology, The Second Xiangya Hospital, Central South University, Changsha, Hunan, China; Sapporo Ika Daigaku, JAPAN

## Abstract

The Akt (protein kinase B)/mammalian target of rapamycin (mTOR) pathway, which is dysregulated in various cancers, controls the assembly of eukaryotic translation initiation factor 4F (eIF4E) complex. However, whether aberrant expression of phosphorylated Akt (p-Akt), phosphorylated mTOR (p-mTOR) and phosphorylated eIF4E (p-eIF4E) is associated with clinicopathological characteristics in surgically resected non-small cell lung cancer (NSCLC) has been rarely reported. Here, we investigated expression of p-Akt, p-mTOR and p-eIF4E proteins in NSCLC by immunohistochemistry and evaluated their correlation with clinicopathological characteristics and prognostic significance. The results showed that the positive percentage of p-Akt, p-mTOR and p-eIF4E was higher in NSCLC. Additionally, p-mTOR and p-eIF4E was dramatically higher in lung adenocarcinoma (both *P*<0.05). Most importantly, NSCLC patients with lymph node metastasis had significantly elevated expression of p-Akt, p-mTOR and p-eIF4E (all *P*<0.05). Positive expression of p-Akt, and any positive expression of p-Akt, p-mTOR and p-eIF4E proteins were positively correlated with clinical stages (both *P*<0.05). Spearman’s rank correlation test revealed that expression of p-Akt was correlated with p-eIF4E and p-mTOR (r = 0.107, *P* = 0.047; r = 0.287, *P*<0.001, respectively). Also, p-eIF4E had positive correlation with p-mTOR (r = 0.265, *P*<0.001). Furthermore, NSCLC patients with increased expression of p-Akt, p-mTOR and p-eIF4E, and any positive expression of above three proteins had lower overall survival rates (all *P*<0.05). Multivariate Cox regression analysis further indicated thatp-eIF4E was an independent prognostic factor for NSCLC patients (*P* = 0.046). Taken together, overexpression of p-Akt, p-mTOR and p-eIF4E proteins is associated with metastasis and poor prognosis of NSCLC patients after surgical resection, and positive expression of p-eIF4E protein may act as an independent unfavorable prognostic biomarker for overall survival of NSCLC patients.

## Introduction

Lung cancer is the most common cancer and the leading cause of cancer related death worldwide [1]. As the most common subtype, non-small cell lung cancer (NSCLC) accounts for 85–90% of all lung cancers [2]. Compared to surgery, adjuvant chemotherapy and targeted therapy are the most effective approaches for the patients with advanced-stage NSCLC [3]. However, most patients with advanced NSCLC have an unfavorable prognosis [4]. Therefore, it is crucial to develop more effective and specific therapy by studying precise molecular mechanism and searching new therapeutic targets and prognosis markers of NSCLC.

The PI3K/AKT/mTOR axis involved in tumor survival, proliferation and distant metastasis and the relevant targeted therapy is under study [5–6]. Akt, the crucial substrate of PI3K, is predominately phosphorylated and activated by PI3K and mammalian target of rapamycin complex 2 (mTORC2), which regulates various biological functions, including cell growth, survival and invasion [7–8]. Consecutively, activated Akt can phosphorylate numerous downstream molecules including mammalian target of rapamycin complex 1 (mTORC1). Subsequently, mTORC1 phosphorylates eIF4E-binding proteins (4E-BPs) and (p70 ribosomal protein S6 kinase) p70S6K [9–10]. Phosphorylation of 4E-BPs dissociates from eIF4E and available eIF4E facilitates its assembly into eukaryotic initiation factor 4F (eIF4F) complex [11–12]. Meanwhile, eIF4E can also be regulated by mitogen-activated protein kinase (MAPK) pathways, which is involved in the development and progression of tumor [13]. Akt/mTOR signaling cascade regulates cell proliferation, survival, metabolism as well as angiogenesis [14]. Activation of Akt/mTOR signal axis plays a crucial role in malignant progression of various malignant tumors, including nasopharyngeal carcinoma, glioblastoma and synovial sarcoma [15–19]. The correlation between phosphorylation level of Akt, mTOR and eIF4E proteins and clinicopathological characteristics, and their prognostic significance in surgically resected NSCLC is rarely reported.

In this study, we investigated the association between expression of p-Akt, p-mTOR and p-eIF4E proteins and clinicopathological characteristics in 341 cases of NSCLC and 91 cases of non-cancer lung tissues by immunohistochemistry (IHC) using high-throughput tissue microarray. Our data indicated that elevated expression of p-Akt, p-mTOR and p-eIF4E proteins associated with metastasis and poor overall survival in NSCLC patients after surgical resection, positive expression of p-eIF4E protein was revealed as an independent unfavorable prognostic biomarker for overall survival of NSCLC patients.

## Materials and methods

### Ethics statement

Samples were obtained with informed consent and all protocols were approved by the Ethics Review Committee of the Second Xiangya Hospital of Central South University (Scientific and Research Ethics Committee, No: S039/2011). Written informed consent was obtained from all patients, and the written informed consent was obtained from the next of kin, caretakers, or guardians on the behalf of the minors/children participants involved in the study.

### Tissue Samples and clinical data

All samples were obtained from the tissue archives of the Pathology Department, the Second Xiangya Hospital of Central South University (Changsha, China) from January 2006 to December 2015. The diagnoses of all cases were confirmed by an expert pathology review (SF). In this study, two groups of specimens were evaluated: 341 samples of primary NSCLC, which included 159 (46.6%) samples of lung squamous cell carcinoma (SCC), 182 (53.4%) samples of lung adenocarcinoma (ADC) and 91 samples of non-neoplastic lung tissue from patients with chronic lung disease. The number of cases in this article was different from the previous published article [20] because only a few samples were selected in the previous article. All NSCLC patients were diagnosed according to the 2015 WHO classification of Lung Tumours, and the TNM stage classification was carried out based on the eighth edition Lung Cancer [21]. High-throughput tissue microarrays (TMAs) were designed and constructed according to the protocol described in previous [22]. The patient demographic and basic clinicopathlogical features were summarized in S1 Table. In this study, we chose the cutoff of age (<55, ≥55) based on the average age of these patients. Overall survival time was calculated from the date of the primary tumor resection to the death due to NSCLC (or the last observation point). All patients received no treatment before the operation.

### Immunohistochemistry (IHC) staining and assessment

The IHC staining was performed by using ready-to-use Envision TM^+^ Dual Link System-HRP methods (Dako; Carpinteria, CA) according to the protocol described in previous [16, 23–25]. The primary antibodies were used as follows: 1:100 dilution of the primary antibody to p-Akt (S473) (Catalog: #2118–1, Epitomics), 1:100 dilution of the primary antibody to p-mTOR (Ser2448) (Catalog: #2976, Cell Signaling) and 1:500 dilution of the primary antibody to p-eIF4E (S209) (Catalog: #2227–1, Epitomics). In addition to the internal positive control, positive control slides were used in each experiment. For negative control, the primary antibody was replaced with IgG isotype-matched antibody.

Immunohistochemical scores were evaluated independently by two experienced pathologists (QW and JF) who did not know the patient characteristic. Positive expression of p-Akt was located in the cytoplasm and nuclear or cell membrane, while p-mTOR and p-eIF4E were shown in the cytoplasm of NSCLC and non-cancerous lung tissue. Evaluation based on the references for the expression of p-Akt, p-mTOR and p-eIF4E proteins was described as follows [16, 23–25]: Staining intensity for p-Akt, p-mTOR and p-eIF4E was classified as 0 (no staining), 1 (weak staining), 2 (moderate staining), and 3 (strong staining). The positive percentage was categorized as 0 (0%), 1 (1%-20%), 2 (21%-50%), 3 (51%-75%), and 4 (76%-100%). the final score was determined by multiplication of two criteria mentioned above, and ranged from 0–12. score ≤1 was regarded as low expression and >1 was regarded as high expression. Agreement between the two evaluators was 95%, and all scoring differences were resolved through discussion.

### Statistical analysis

SPSS version 24.0 (SPSS, Chicago, IL) was used for all statistical analyses in this study. Chi-square test was performed to evaluate the relationship between expression of p-Akt, p-mTOR and p-eIF4E proteins and clinicopathological characteristics. Spearman rank correlation coefficient was hired to assess correlations among these three proteins in NSCLC. Overall survival probabilities were investigated by Kaplan-Meier analysis and the statistical significance was estimated using the log-rank test. Cox proportional hazard regression analysis was utilized to determine the independent prognostic markers for NSCLC patients among clinicopathological characteristics and expression of these three proteins. All *P*-values in this study were two-sided statistical analysis, and the differences were regarded as statistically significant when *P* value was less than 0.05.

## Results

### High expression of p-Akt, p-mTOR and p-eIF4E proteins in NSCLC

**Fig 1:**
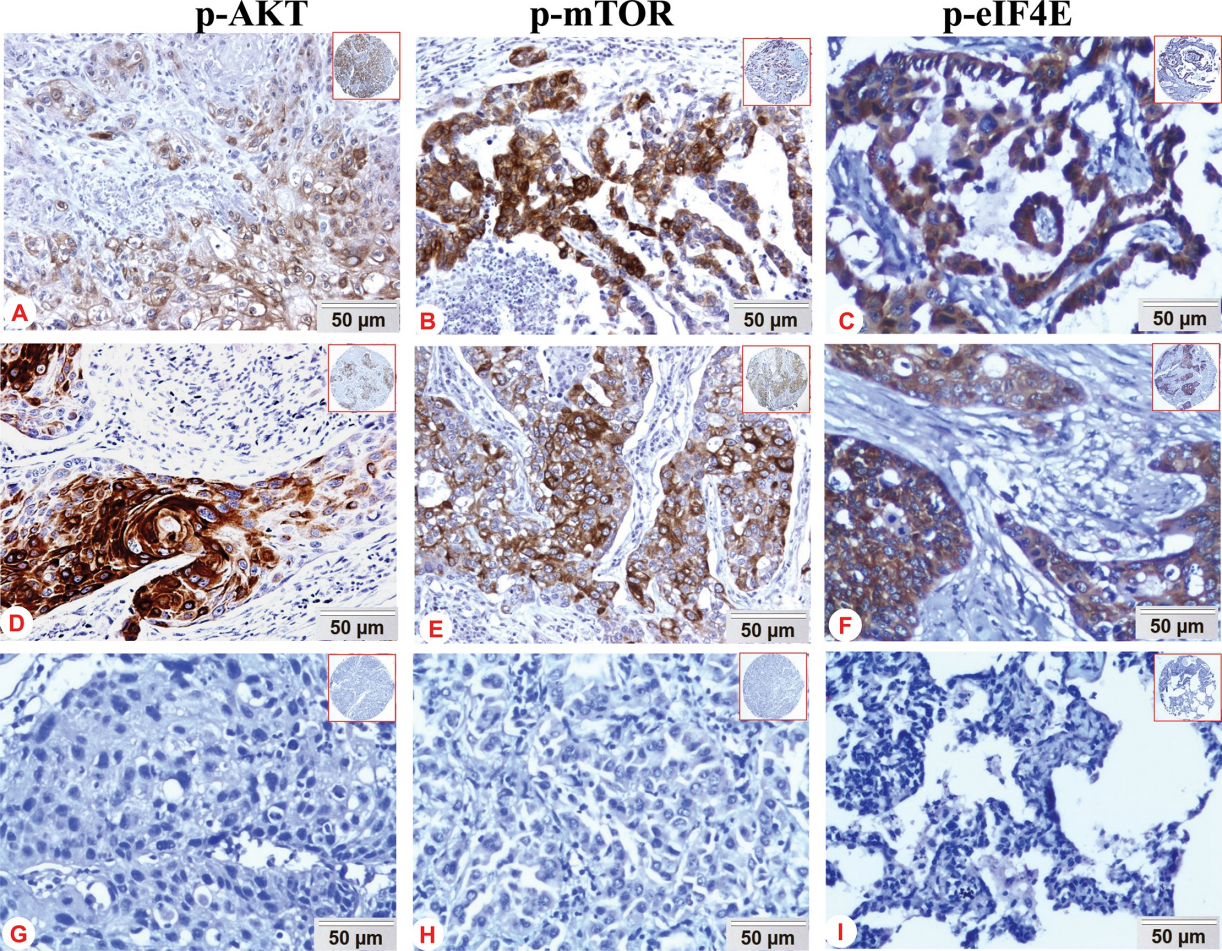
Expression of p-Akt, p-mTOR and p-eIF4E proteins in NSCLC and non-cancerous lung tissue was detected by immunohistochemistry. Positive expression of p-Akt was mainly located in the cytoplasm, a few in nucleus or membrane of lung ADC (Fig 1A) and lung SCC (Fig 1D); and no positive staining of IgG isotype-matched control antibody in lung SCC (Fig 1G); positive expression of p-mTOR was indicated mainly in the cytoplasm of lung ADC (Fig 1B) and lung SCC (Fig 1E); and negative expression of p-mTOR protein in lung ADC (Fig 1H); positive expression of p-eIF4E was predominantly located in the cytoplasm of lung ADC (Fig 1C) and lung SCC (Fig 1F); no positive expression of p-eIF4E protein in non-cancerous lung tissue (Fig 1I) (DAB staining, magnification 200×).

**Fig 2:**
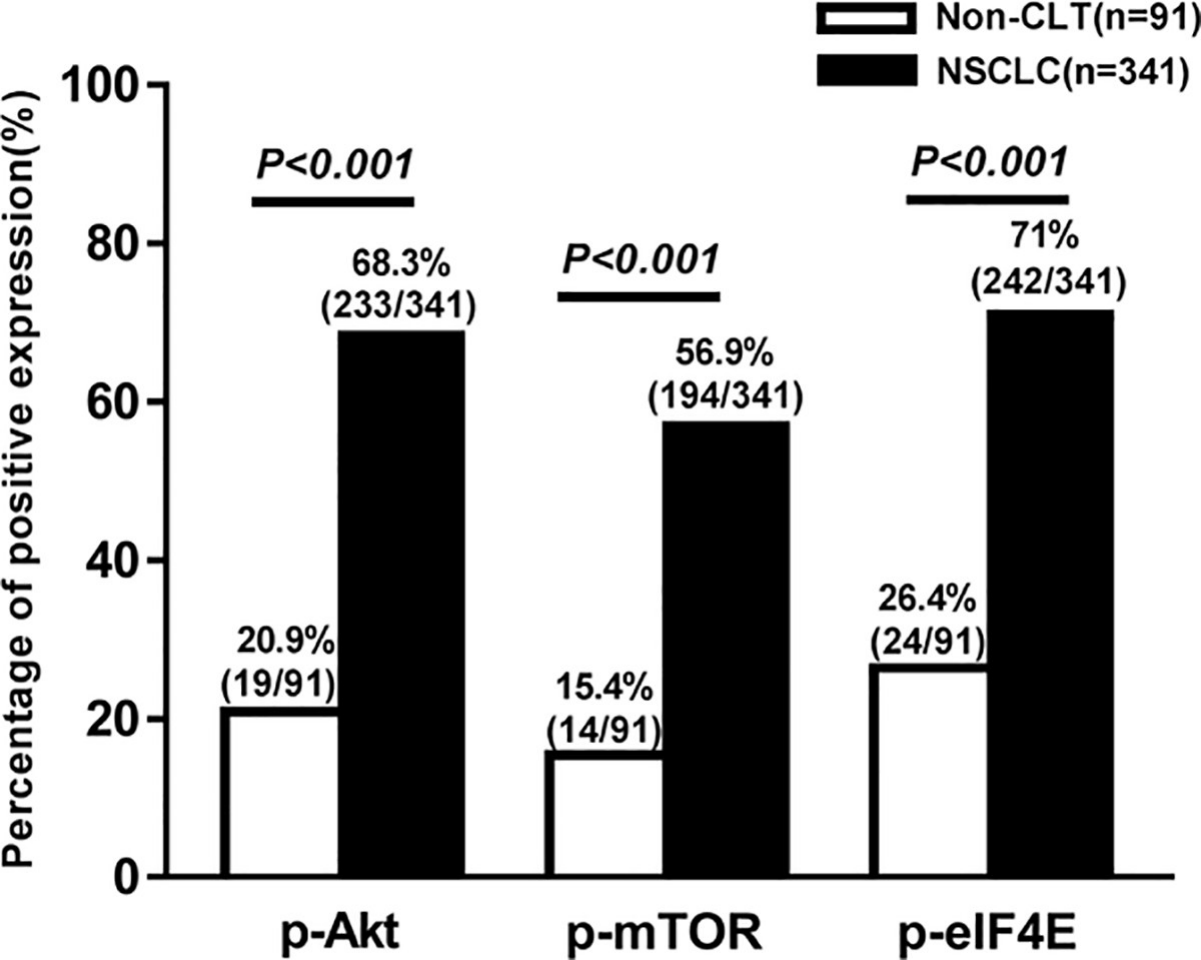
The comparison of expression of p-Akt, p-mTOR and p-eIF4E proteins in NSCLC and non-cancerous lung tissue (Non-CLT). Results showed that positive expression of p-Akt, p-mTOR and p-eIF4E proteins was significantly higher in NSCLC than that in the Non-CLT (*P*<0.001).

Expression and cellular localization of p-Akt, p-mTOR, and p-eIF4E proteins in NSCLC and the non-cancerous lung tissues were examined by IHC. The positive expression of p-Akt protein was mainly located in the cytoplasm, a few in nuclear or membrane of lung ADC (Fig 1A) and lung SCC (Fig 1D), and no positive staining of IgG isotype-matched control antibody was found in lung SCC (Fig 1G). In addition, positive staining of p-mTOR (Fig 1B–1E) and p-eIF4E (Fig 1C–1F) was shown in the cytoplasm of the lung ADC and lung SCC, and there was negative expression of p-mTOR protein in lung ADC (Fig 1H). Also, no positive staining of p-eIF4E was shown in non-cancerous lung tissue (Fig1I). The positive percentage of p-Akt, p-mTOR, and p-eIF4E were 68.3% (233/341), 56.9% (194/341), 71% (242/341) in NSCLC, respectively, whereas 20.9% (19/91), 15.4% (14/91), and 26.4% (24/91) in non-cancerous lung tissues (Non-CLT), respectively. The expression of p-Akt, p-mTOR, and p-eIF4E between NSCLC and Non-CLT group had statistical significance (*P*<0.001 for all) (Fig 2).

### Analysis of associations between the expression of p-Akt, p-mTOR and p-eIF4E proteins and clinicopathological characteristics in NSCLC

We further investigated the associations between the expression of p-Akt, p-mTOR and p-eIF4E proteins and clinicopathological characteristics of NSCLC. As shown in Table 1, positive expression of p-Akt and any positive of p-Akt, p-mTOR and p-eIF4E proteins in the smoker NSCLC patients was statistically higher than those in the never-smoker patients (*P* = 0.022, *P* = 0.004, respectively). There was significantly higher positive expression of p-mTOR (*P*<0.001) and p-eIF4E (*P* = 0.002) in lung ADC compared to lung SCC. Positive percentage of p-Akt and any positive of p-Akt, p-mTOR and p-eIF4E proteins expression were positively associated with clinical stages of NSCLC patients (*P* = 0.023, *P* = 0.035, respectively). Most importantly, positive expression of p-Akt, p-mTOR and p-eIF4E in NSCLC patients with lymph node metastasis (LNM) was significantly higher than those without LNM (*P* = 0.033, *P* = 0.025 and *P* = 0.036, respectively). Also, the results showed that positive expression of p-eIF4E was frequently higher in poor differentiated NSCLC compared with well differentiated NSCLC (*P* = 0.020). In addition, high expression of p-mTOR protein was revealed in the female NSCLC patients (*P* = 0.001). However, no significant differences were shown between the expression of p-Akt and any positive of above three proteins and histological type (*P*>0.05 for all). Also, no correlation was observed between the expression of p-Akt, p-mTOR and p-eIF4E proteins and any positive of above three proteins and NSCLC patient’s age (*P*>0.05 for all). There was no association between positive expression of p-Akt and p-eIF4E proteins and gender, No correlation was observed between expression of p-Akt and histological type and pathological grade of NSCLC (*P*>0.05 for all). All data were detailed in Table 1.

**Table 1:** Analysis of the association between the expression of p-Akt, p-mTOR and p-eIF4E proteins and clinicopathological characteristics of NSCLC (n = 341).

Clinicopathological characteristics	p-Akt			p-mTOR			p-eIF4E			p-Akt/p-mTOR/p-eIF4E		
	P (%)	N (%)	*P*-value	P (%)	N (%)	*P*-value	P (%)	N (%)	*P*-value	P^+^	N^-^	*P*-value
**Age (years)**												
<55	96(70.1)	41(29.9)		78(56.9)	59(43.1)		94(68.6)	43(31.4)		128(93.4)	9(6.6)	
≥55	137(67.2)	67(32.8)	0.570	116(56.9)	88(43.1)	0.990	148(72.5)	56(27.5)	0.432	180(88.2)	24(11.8)	0.112
**Gender**												
Male	177(69.1)	79(30.9)		133(52)	123(48.0)		179(69.9)	77(30.1)		231(90.2)	25(9.8)	
Female	56(65.9)	29(34.1)	0.576	61(71.8)	24(28.2)	0.001*	63(74.1)	22(25.9)	0.460	77(90.6)	8(9.4)	0.924
**Smoking status**												
Smoker	89(60.5)	58(39.5)	0.022*	83(56.5)	64(43.5)	0.889	101(68.7)	46(31.3)	0.423	125(85.0)	22(15.0)	0.004*
Non-smoker	144(49.0)	50(51.0)		111(57.2)	83(42.8)		141(72.7)	53(27.3)		183(94.3)	11(5.7)	
**Histological type**												
ADC	117(64.3)	65(35.7)		133(73.1)	49(26.9)		142(78.0)	40(22.0)		168(92.3)	14(7.7)	
SCC	116(73.0)	43(27.0)	0.086	61(38.4)	98(61.6)	0.000*	100(62.9)	59(37.1)	0.002*	140(88.1)	19(11.9)	0.185
**Clinical stages**												
Stage I and II	105(62.5)	63(37.5)		87(51.8)	81(48.2)		113(67.3)	55(32.7)		146(86.9)	22(13.1)	
Stage III	128(74.0)	45(26.0)	0.023*	107(61.8)	66(38.2)	0.061	129(74.6)	44(25.4)	0.137	162(93.6)	11(6.4)	0.035*
**LN status**												
No LNM	86(61.9)	53(38.1)		69(49.6)	70(50.4)		90(64.7)	49(35.3)		121(87.1)	18(12.9)	
LNM	147(72.8)	55(27.2)	0.033*	125(61.9)	77(38.1)	0.025*	152(75.2)	50(24.8)	0.036*	187(92.6)	15(7.4)	0.090
**Histological grades**												
Well and moderate	103(66)	53(34.0)		80(51.3)	76(48.7)		101(64.7)	55(35.3)		137(87.8)	19(12.2)	
Poor	130(70.3)	55(29.7)	0.401	114(61.6)	71(38.4)	0.055	141(76.2)	44(23.8)	0.020*	171(92.4)	14(7.6)	0.151

Abbreviations: ADC: lung adenocarcinoma, SCC: lung squamous carcinoma, LN: lymph node, LNM: lymph node metastasis

*: statistically significant (p < 0.05), statistical analysis was performed P^+^: positive expression of any positive of p-Akt, p-mTOR and p-eIF4E; N^-^: common negative staining of p-Akt, p-mTOR and p-eIF4E

### The pairwise correlation between the expression of p-Akt, p-mTOR and p-eIF4E proteins in 341 cases of NSCLC

Spearman’s rank correlation test was utilized to investigate the pairwise correlation among positive expression of p-Akt, p-mTOR and p-eIF4E proteins in NSCLC. Table 2 showed that p-Akt had a positive correlation with p-mTOR (r = 0.107, *P* = 0.047) and p-eIF4E (r = 0.287, *P*<0.001) in NSCLC. Meanwhile, there was an evidently positive correlation between the expression of p-mTOR and p-eIF4E protein in the NSCLC (r = 0.265, *P*<0.001).

**Table 2:** The pairwise association between expression of p-Akt, p-mTOR and p-eIF4E proteins in 341 cases of NSCLC.

	p-Akt	p-mTOR	p-eIF4E
p-Akt			
Spearman’s correlation coefficient	1	0.107	0.287
Sig. (2-tailed)	-	0.047*	<0.001**
p-mTOR			
Spearman’s correlation coefficient	0.107	1	0.265
Sig. (2-tailed)	0.047*	-	<0.001**
p-eIF4E			
Spearman’s correlation coefficient	0.287	0.265	1
Sig. (2-tailed)	<0.001**	<0.001**	-

NOTE. Values are Spearman's correlation coefficient

* Correlation is significant at the *P* < 0.05 level (2-tailed)

** Correlation is significant at the *P* < 0.01 level (2-tailed).

### Positive expression of p-Akt, p-mTOR and p-eIF4E proteins inversely related to the overall survival rates of NSCLC patients

**Fig 3:**
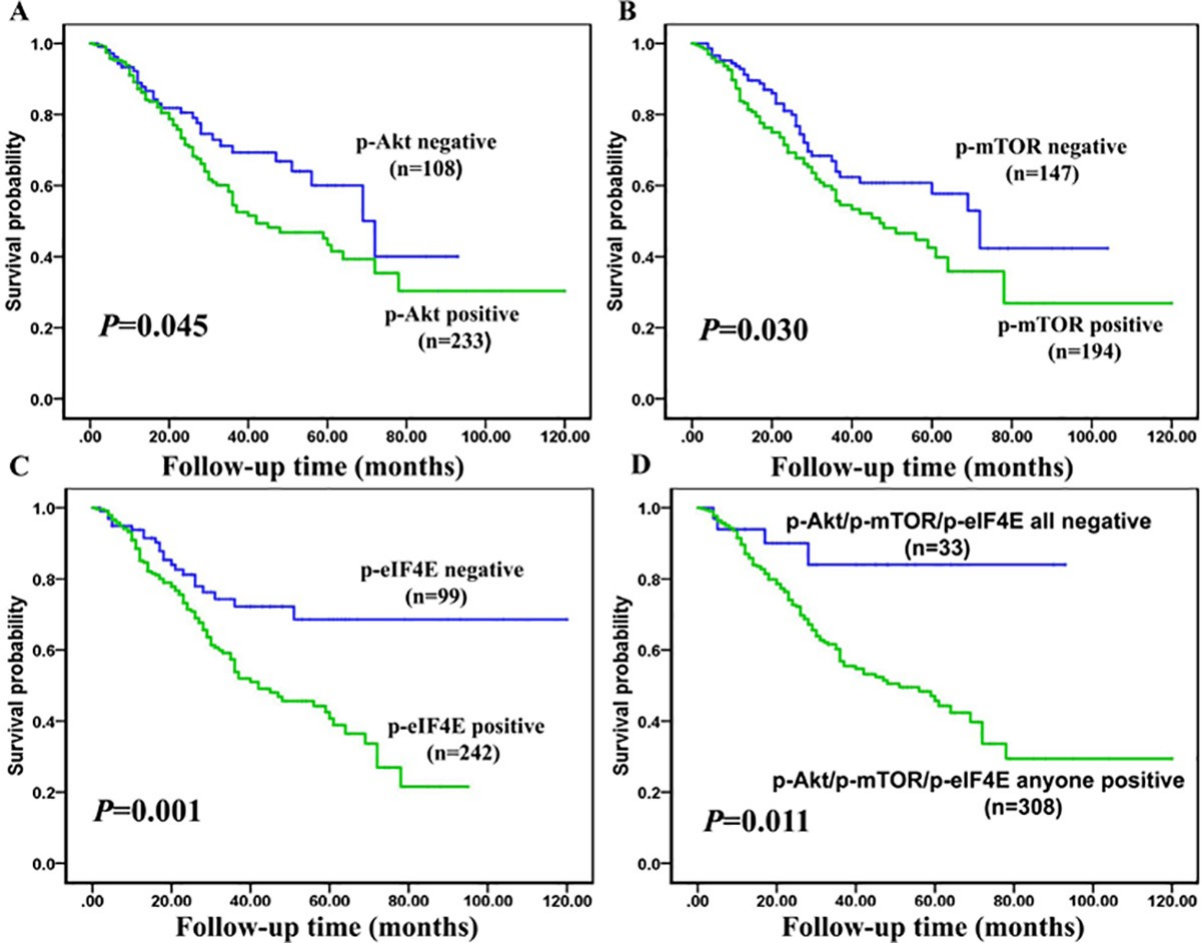
Kaplan-Meier analysis to plot the survival curve of NSCLC patients with expression of p-Akt, p-mTOR and p-eIF4E proteins and statistical significance were statistically evaluated by the log-rank test. Fig 3A: There was worse overall survival for NSCLC patients with p-Akt positive expression compared to patients with p-Akt negative staining (*P* = 0.045, two sided). Fig 3B: Result showed that statistically significant shorter overall survival for NSCLC patients with expression of p-mTOR protein compared to patients with p-mTOR negative expression (*P* = 0.030, two sided). Fig 3C: Kaplan-Meier curves indicated significantly shorter overall survival for NSCLC patients with expression of p-eIF4E compared to patients with negative expression of p-eIF4E (*P* = 0.001, two sided). Fig 3D: Kaplan-Meier curves also revealed worse overall survival for NSCLC patients with expression of any positive of p-Akt, p-mTOR and p-eIF4E proteins compared to patients with common negative staining of three proteins above (*P* = 0.011, two sided).

Fig 3 showed the Kaplan-Meier survival plots for NSCLC patients with positive expression of p-Akt, p-mTOR and p-eIF4E proteins and any positive expression of three proteins above. The overall survival rates (OS) of NSCLC patients with positive expression of p-Akt, p-mTOR and p-eIF4E proteins were significantly lower than those with negative expression (*P* = 0.045, Fig 3A; *P* = 0.030, Fig 3B; and *P* = 0.001, Fig 3C, respectively). More strikingly, the NSCLC patients with any positive of p-Akt, p-mTOR and p-eIF4E proteins had shorter OS than those with all negative expression (*P* = 0.011, Fig 3D).

Furthermore, multivariate Cox proportional hazard regression analysis was applied to evaluate whether the expression of p-Akt, p-mTOR and p-eIF4E proteins was the independent unfavorable prognostic biomarkers for overall survival of NSCLC patients. Multivariate analysis of variables containing clinical stages, LNM status, histological types, pathological grades, age, gender, smoking status and expression of p-Akt, p-mTOR and p-eIF4E proteins was presented in Table 3. Results indicated that positive expression of p-eIF4E protein can be considered as an independent poor prognostic factor for NSCLC patients (*P* = 0.046), as well as clinical stages (*P* = 0.003) and LNM (*P* = 0.028). However, no significant importance was discovered in age, gender, histological types, and pathological grades of the NSCLC patients, as well as expression of p-mTOR and p-Akt proteins (*P*>0.05 for all).

**Table 3:** Summary of multivariate statistical analysis of p-Akt, p-mTOR and p-eIF4E protein expression for overall survival rates in NSCLC patients (n = 341).

Variables	SE	Wald	Sig.	Exp(B)	95.0% CI for Exp(B)	
					Lower	Upper
**Age**	.189	.025	.875	.971	.670	1.405
**Gender**	.234	2.510	.113	.690	.437	1.092
**Smoking status**	.212	3.465	.073	1.407	.952	2.179
**LNM status**	.223	4.802	.028*	1.630	1.053	2.522
**Histological type**	.217	.000	.990	.997	.651	1.527
**Pathological grades**	.193	3.413	.065	1.429	.978	2.088
**Clinical stages**	.218	8.594	.003*	1.894	1.236	2.903
**p-Akt**	.225	.247	.620	1.118	.719	1.739
**p-mTOR**	.215	1.461	.227	1.297	.851	1.978
**p-eIF4E**	.249	3.984	.046*	1.642	1.009	2.673

Abbreviations: LNM, lymph node metastasis; SE, standard error (SE); Wald, wald test; Sig, significance; Exp (B), exponentiation of the B coefficient; CI, confidence interval.

Note: multivariate analysis of Cox proportional hazard regression

*P<0.05.

## Discussion

Akt is a major protein in the PI3K-AKT-mTOR pathway, which is also a well-recognized proto-oncogene. Received stimulation from PI3K and mTORC2, activated Akt consecutively phosphorylates mTOR, which is a master regulator in translation and gene expression [26]. Previous studies indicated that p-Akt is elevated in a range of malignancies (e.g., breast, kidney, bladder cancer and lung cancer) and is significantly associated with unfavorable prognosis [27–30]. But in our previous study of nasopharyngeal carcinoma (NPC), we found that p-Akt protein was not related to prognosis, while p-p70S6K and p-4EBP1 proteins were significantly correlated with prognosis [16]. However, in our current study of NSCLC, we found that p-Akt, p-mTOR and p-eIF4E were all related to prognosis, which needs to be further proved. Furthermore, p-Akt can induce radiation resistance and be positively correlated with recurrence in cervical cancer [31]. In our large samples research, NSCLC had evidently elevated expression of p-Akt protein, which further proves the previous results. Positive expression of p-Akt and any positive of p-Akt/p-mTOR/ p-eIF4E were statistically higher than those in the never-smoker patients. Moreover, positive expression of p-Akt protein was more frequent in advanced NSCLC patients, while p-Akt protein was not higher in advanced stage of NPC and esophageal squamous cell carcinoma (ESCC) [32–33]. Also, NSCLC patients with positive expression of p-Akt proteins tend to have LNM and lower overall survival rates. All these data might identify that high expression of p-Akt protein had clinical and prognostic significance in the NSCLC.

Stimulated by hormones and growth factors, mTORC1 subsequently activates two critical substrates named 4E-BPs and p70S6K. Activated 4E-BPs being dissociated from eIF4E enables the assembly of eIF4F complex, which initiates the translational processing. P70S6K promotes the phosphorylation of eIF4B and (programmed cell death 4) PDCD4, which leads to mRNA translation [34]. As the conjunction of Akt/mTOR and MAPK signal axis, eIF4E can also be activated through the MNK protein kinases [13]. Previous study has shown that mTOR and eIF4E are two major proto-oncogenes related to oncogenic transformation, which are involved in tumor growth and development [12, 35]. An investigation has shown that high expression of p-mTOR is correlated with presence of LNM and advanced stage of penile squamous cell carcinoma [36]. Also, expression level of p-mTOR is inversely associated with disease-free survival time of gastric cancer [37].

In our study, we found that NSCLC patients had high positive expression of p-mTOR and p-eIF4E proteins, which supports our previous findings in NPC and astrocytomas [38–40]. Expression of p-Akt was mainly localized in cytoplasm, a few in the nuclear or membrane, which indicates that p-Akt may play an important role in the cytoplasm and nucleus/membrane, thereby activating its downstream signaling pathway and promoting the development of tumors. Also, there was significantly higher positive expression of p-mTOR and p-eIF4E proteins in lung ADC than that in lung SCC, which might indicate the p-mTOR and p-eIF4E were associated with histological type and could be used to improve the IHC staining algorithm currently used to sub-classify NSCLC into lung adenocarcinomas versus lung squamous subtypes. More strikingly, elevated expression of p-mTOR and p-eIF4E proteins positively correlated NSCLC with LNM, which was also found in NPC and further proved that p-eIF4E protein might promote metastasis [38]. Positive expression of p-eIF4E protein was frequently observed in NSCLC with poor differentiation. What’s more, there were positive associations between expression of p-Akt and p-mTOR, p-Akt and p-eIF4E, p-eIF4E and p-mTOR in the NSCLC. These findings suggested that p-Akt, p-mTOR and p-eIF4E proteins were mutually regulated in the development of NSCLC. Finally, NSCLC patients with positive expression of p-Akt, p-mTOR and p-eIF4E proteins and any positive of p-Akt, p-mTOR and p-eIF4E proteins had poorer overall survival rate compared with those with all negative expression. Furthermore, positive expression of p-eIF4E was identified as independent poor prognostic factor in NSCLC regardless of clinical stages and LNM, which was in favor of previous studies [38, 40–41]. Our findings deepened the understanding of the significance of p-Akt, p-mTOR and p-eIF4E proteins expression in NSCLC by jointly exploring in a large sample.

In summary, our finding showed that elevated expression of p-Akt, p-mTOR and p-eIF4E proteins was evidently associated with metastasis and poor prognosis of NSCLC patients. Moreover, positive expression of p-eIF4E protein might be recognized as a valuable independent unfavorable prognostic biomarker for overall survival of NSCLC patients after surgical resection.

## Supporting information

S1 Table Clinicopathological characteristics of patients with non-small cell lung cancer (NSCLC) and non-cancerous lung tissues in the tissue microarrays. (DOCX)

S1 Data Minimal data set. (XLSX)
