# Supplementary material for: Overexpression of p-Akt, p-mTOR and p-eIF4E proteins associates with metastasis and unfavorable prognosis in non-small cell lung cancer
Source: PLoS One. 2020 Feb 5;15(2):e0227768. doi: 10.1371/journal.pone.0227768 (PMC7001968; doi:10.1371/journal.pone.0227768)
Supplement: S1 Table — (DOCX) [file pone.0227768.s001.docx]

Table S1. Clinicopathological characteristics of patients with non-small cell lung cancer (NSCLC) and non-cancerous lung tissues in the tissue microarrays

| Patients characteristics | No. of patients (%) |
| --- | --- |
| **NSCLC** |  |
| **Age (years)** |  |
| <55 | 137 (40.2) |
| ≥55 | 204 (59.8) |
| **Gender** |  |
| Male | 256 (75.1) |
| Female | 85 (24.9) |
| **Clinical stages** |  |
| Stage I and II | 168 (49.3) |
| Stage III | 173 (50.7) |
| **Lymph node status** |  |
| No LNM | 139 (40.8) |
| LNM | 202 (59.2) |
| **Histological type** |  |
| SCC | 159 (46.6) |
| ADC | 182 (53.4) |
| **Differentiation** |  |
| Well and moderate | 156 (45.7) |
| Poor  **Survival status**  Alive  Dead | 185 (54.3)  220 (64.5)  121 (35.5) |
| **Smoking status** |  |
| Smoker | 147 (43.1) |
| Non-smoker | 194 (56.9) |
| **non-cancerous lung tissues** |  |
| **Age(years)** |  |
| <55 | 47 (51.6) |
| ≥55 | 44 (48.4) |
| **Gender** |  |
| Male | 63 (69.2) |
| Female | 28 (30.8) |
| **Smoking status** |  |
| Smoker | 36 (39.6) |
| Non-smoker | 55 (60.4) |
